# Marine Archaeon *Methanosarcina acetivorans* Enhances Polyphosphate Metabolism Under Persistent Cadmium Stress

**DOI:** 10.3389/fmicb.2019.02432

**Published:** 2019-10-24

**Authors:** Ricardo Jasso-Chávez, Elizabeth Lira-Silva, Kasia González-Sánchez, Violeta Larios-Serrato, Diana Lucía Mendoza-Monzoy, Fernando Pérez-Villatoro, Enrique Morett, Alicia Vega-Segura, M. Eugenia Torres-Márquez, Armando Zepeda-Rodríguez, Rafael Moreno-Sánchez

**Affiliations:** ^1^Departamento de Bioquímica, Instituto Nacional de Cardiología, Mexico City, Mexico; ^2^Departamento de Farmacología, Instituto Nacional de Cardiología, Mexico City, Mexico; ^3^Winter Genomics, Mexico City, Mexico; ^4^Instituto Nacional de Medicina Genómica, Mexico City, Mexico; ^5^Instituto de Biotecnología, UNAM, Cuernavaca, Mexico; ^6^Facultad de Medicina, UNAM, Mexico City, Mexico

**Keywords:** archaeal metabolism, heavy metal-binding molecules, polyphosphate kinase, exopolyphosphatase, biofilm induction, methanogenesis

## Abstract

Phosphate metabolism was studied to determine whether polyphosphate (polyP) pools play a role in the enhanced resistance against Cd^2+^ and metal-removal capacity of Cd^2+^-preadapted (CdPA) *Methanosarcina acetivorans*. Polyphosphate kinase (PPK), exopolyphosphatase (PPX) and phosphate transporter transcript levels and their activities increased in CdPA cells compared to control (Cnt) cells. K^+^ inhibited recombinant Ma-PPK and activated Ma-PPX, whereas divalent cations activated both enzymes. Metal-binding polyP and thiol-containing molecule contents, Cd^2+^-removal, and biofilm synthesis were significantly higher in CdPA cells >Cnt cells *plus* a single addition of Cd^2+^>Cnt cells. Also, CdPA cells showed a higher number of cadmium, sulfur, and phosphorus enriched-acidocalcisomes than control cells. Biochemical and physiological phenotype exhibited by CdPA cells returned to that of Cnt cells when cultured without Cd^2+^. Furthermore, no differences in the sequenced genomes upstream and downstream of the genes involved in Cd^2+^ resistance were found between CdPA and Cnt cells, suggesting phenotype loss rather than genome mutations induced by chronic Cd^2+^-exposure. Instead, a metabolic adaptation induced by Cd^2+^ stress was apparent. The dynamic ability of *M. acetivorans* to change its metabolism, depending on the environmental conditions, may be advantageous to remove cadmium in nature and biodigesters.

## Introduction

Inappropriate treatment of water polluted with heavy metals has become a public health problem around the world. Cadmium is found in soils, oceans, lakes, rivers, and other water bodies as well as in sewage sludge (Ahring and Westermann, 1985; Zheng-Bo et al., 2007; Altaş, 2009). Some plants and microorganisms have demonstrated a remarkable ability to remove Cd^2+^ from soils and water, and hence they may be considered realistic models for bioremediation (Leung et al., 2000; García-García et al., 2016; Moreno-Sánchez et al., 2017).

Organisms from the *Archaea*, known to inhabit extreme environments, have also been detected in environments with high heavy metal concentrations. The presence of methanogenic archaea in marine sediments polluted by Cd^2+^, Cu^2+^, Pb^2+^, and Hg^2+^ (Almeida et al., 2009) has led to the hypothesis that archaea have developed mechanisms to cope with heavy metal stress derived from geological events. These self-protective mechanisms now should enable them to counter the elevated anthropogenic-derived pollution. Transcriptomic and proteomic studies have described these mechanisms in archaea. However, biochemical characterization has yet to be elucidated (Mangold et al., 2013; Almárcegui et al., 2014; McCarthy et al., 2014).

Resistance mechanisms against heavy metals (Cu^2+^ and Cd^2+^) in archaea are similar to those found in eubacteria and eukaryotes. Thus far, they are extrusion by cation diffusion facilitators or heavy metal-specific ATP-dependent pumps; external binding by extracellular polymeric substances; biotransformation/inactivation by chemical reduction; and intracellular inactivation by chelating agents (Spada et al., 2002; Alvarez and Jerez, 2004; Baker-Austin et al., 2005; Harris et al., 2005; Remonsellez et al., 2006; Focardi et al., 2012; Mangold et al., 2013; Das et al., 2014; Singh et al., 2015).

*Methanosarcina acetivorans* C2A is a marine archaeon with the ability to accumulate Cd^2+^ by coupling it with sulfur-containing molecules (Lira-Silva et al., 2012). Cd^2+^ stress induces an increase of Cys, S^2–^ and the methyl carrier coenzyme M intracellular contents (CoM-SH). However, thiol-containing molecules were not enough to bind and inactivate all Cd^2+^ (Lira-Silva et al., 2013). Moreover, *M. acetivorans* strain (CdPA) tolerates up to 2 mM total external Cd^2+^ (0.8 mM free Cd^2+^) after chronic pre-exposure to low (50 μM) Cd2+(Lira-Silva et al., 2013). Other CdPA cell features might contribute to enhanced heavy metal resistance/accumulation. Higher capacity of biofilm formation may trap a high percentage of Cd^2+^ outside the cells. Their increased levels of total orthophosphate (Pi), PPi and polyphosphate (polyP) may trap more Cd^2+^ inside the cells (Jasso-Chávez et al., 2015). Indeed, it has been described that polyP increases under stress conditions in *Escherichia coli* and *M. acetivorans* (Keasling and Hupf, 1996; Jasso-Chávez et al., 2015). Methanogens have also shown ability to contend against other heavy metals like uranium (Holmes et al., 2018) and mercury (Gilmour et al., 2018). Therefore, methanogens emerge as a suitable model that may help to understand the interactions between archaea and the increasing presence of heavy metal in their ecological niches.

To unveil whether chronic exposure to Cd^2+^ may induce permanent or transitory changes in polyP metabolism, in the present work the genome of CdPA and their control cells were sequenced and compared with the previously reported genome (Galagan et al., 2002) which was taken as the reference (NCBI ID: 1072). Also, the transcript contents of the *ppk*, *ppx*, and *pstA* genes, PPK and PPX activities, and thiol- and poly phosphate-molecule levels were analyzed. Kinetic characterization of the PPK and PPX recombinant enzymes was also carried out. Data suggested that the chronic Cd^2+^ stress did not induce mutations in the genes involved in metal resistance; instead, fast and transitory metabolic shifts were in polyP, thiol-molecule synthesis and the capacity to synthesize biofilm. This metabolic strategy seemed plentiful to manage Cd^2+^ toxicity.

## Experimental Procedures

### Materials

ATP and phosphoenolpyruvate were from Roche (Germany). L-cysteine, NADH, PK/LDH mix and toluidine blue were purchased from Sigma (United States). Trimethylsilyl polyphosphate was from Sigma-Aldrich (Switzerland). Standard solutions of CdCl_2_ and monobasic potassium phosphate (KH_2_PO_2_) were from Sigma-Aldrich (Germany). HEPES was acquired from Research Organics (United States). Absolute methanol, sodium acetate, Na_2_S⋅9 H_2_O, acetic acid and CdCl_2_⋅2.5 H_2_O, were of analytical grade.

### Cell Growth

*Methanosarcina acetivorans* C2A was cultured in absence (control cells) or presence of 50 μM CdCl_2_, under anoxic conditions in high salt (HS) medium supplemented with 100 mM methanol or acetate as carbon source (Sowers et al., 1993). Cd^2+^-preadapted cells (CdPA) were generated by culturing C2A cells in media containing 50 μM CdCl_2_ and sub-culturing them in fresh 50 μM CdCl_2_ containing medium every 2 weeks for more than 1 year as previously reported (Lira-Silva et al., 2013). Cultures were carried out in 100 mL anaerobic bottles by adding 0.5–0.7 mg cell protein into 50 mL of growth medium, sealed with rubber septa and aluminum crimps. Cell growth was determined by their methane production and protein content. Optical density was not considered as a reliable cell growth parameter, since Cd^2+^ induced biofilm formation also brought about significant turbidity. To determine whether chronic Cd^2+^ stress also conferred resistance toward other heavy metals, Cd^2+^ was replaced by CuSO_4_ or ZnCl_2_ in the culture medium.

### Metabolite Contents

Determination of methane, thiol-containing molecules (Cys, CoM-SH) and sulfide were carried out by gas chromatography, HPLC and spectrophotometry, respectively (Lira-Silva et al., 2013). For extracellular orthophosphate (Pi) quantitation, aliquots of cell-free growth medium withdrawn at the indicated times were assayed by molybdenum blue generation at 870 nm (LeBel et al., 1978). Intracellular polyP content was assessed in cell suspensions previously washed and mixed with 3% (v/v) ice-cold perchloric acid (PCA) as described elsewhere (Jasso-Chávez et al., 2015).

PolyP was co-isolated with DNA as reported (Jasso-Chávez et al., 2017). Thereafter, samples were treated with 1 U of DNAse for 2 h at 37°C and then precipitated with ice-cold absolute ethanol for 1 h at ^–^20°C. The pellet (polyP) was resuspended in 50 mM NaOH + 1 mM EDTA. PolyP samples were mixed with toluidine blue solution and the absorbance measured at 530/630 nm as previously described. PolyP was also identified by 14% urea-PAGE and stained with 0.05% toluidine blue in 25% methanol and 5% glycerol (Gomez Garcia, 2014). Cellular protein precipitated with 10% (w/v) trichloroacetic acid (TCA) overnight, was determined by Lowry method.

### Miscellaneous

Methods regarding the analyses of cell ultrastructure, heavy metal contents and biofilm are described in the Supplementary Text S1.

### Transcript Contents, Cloning and Heterologous Overexpression of the PPX and PPK

Changes in transcript levels were determined by semiquantitative reverse-transcriptase PCR reaction following real time PCR (see Supplementary Table S4 for primers sequences used). mRNA content changes were determined by the 2^–ΔΔCt^ method in CdPA cells or acetate-grown control cells (without Cd^2+^; Cnt), or control cells with a single 50 μM Cd^2+^ exposure (Cnt + Cd), or CdPA cells further cultured without Cd^2+^ for one pass (3–4 generations; CdPA-Cd cells). *gapd* but not MA_3998 gene was used as a house-keeping reference gene (Livak and Schmittgen, 2001; Rohlin and Gunsalus, 2010; Santiago-Martínez et al., 2016; also see Supplementary Text S1 and Supplementary Table S5). Detailed methodology regarding the cloning and heterologous overexpression of recombinant PPX and PPK is described in Supplementary Text S1 (Saavedra et al., 2005; Studier, 2005).

### PPK and PPX Activities Determination

Cytosol-enriched fractions were used to measure enzyme activities and were obtained from cells harvested after 7 or 14 days of growth as described elsewhere (Santiago-Martínez et al., 2016). PPK and PPX activities were determined in the absence and presence of K^+^ using commercial trimethylsilyl polyP. The synthesis or degradation of polyP was determined by mixing the reaction with a dying solution (0.001% toluidine blue in 40 mM acetic acid) and measuring absorbance at 530 and 630 nm (Skorko, 1989; Mullan et al., 2002). The reaction was started by adding the recombinant protein (1.7–4.6 μg protein) or cytosol-enriched fraction (50 μg protein). The reaction mixture containing variable Mg-ATP (PPK) or polyP (PPX) was incubated for 15 or 60 min for PPK or PPX, respectively, at 37°C under 150 rpm orbital agitation. Enzymatic activities were also determined with endogenous polyP-enriched-fractions after incubating for 60 min. See Supplementary Text S1 for details.

### DNA Preparation and Genome Sequencing

Genomic DNA extraction from Cnt- and CdPA-*M. acetivorans* was performed as reported elsewhere (Jasso-Chávez et al., 2017). These strains were sequenced using the Illumina Genome Analyzer IIx platform at the USSDNA (IBT, UNAM) with paired end libraries and 72 base pair reads. FastQC version 0.10.1 and Trimmomatic version 0.32 were used for adaptor sequence removal and quality trimming (Bolger et al., 2014). *De novo* assembly was carried out using ABySS version 1.3.7 (Simpson et al., 2009). For k-mer abundance analysis k-mer version 0.4 was used with a k-mer size of 64. Genome annotation was accomplished using Rapid Annotation using Subsystem Technology (RAST) (Aziz et al., 2008). A comparative analysis was carried out with BLASTN version 2.7.1, using all 4567 coding genes from the reference genome and the strain genomic assemblies. From these datasets, 52 genes associated with putative heavy metal resistance mechanisms were selected for further assessment.

## Results

### Effect of Chronic Cd^2+^ Stress on Cell Growth and Thiol-Metabolite Contents

To assess how the CdPA phenotype develops, an array of biochemical/physiological parameters were compared with control cells (without Cd^2+^; Cnt) and control cells with a single 50 μM Cd^2+^ exposure (Cnt + Cd). To explore phenotype reversibility, CdPA cells were further cultured without Cd^2+^ for one time (3–4 generations; CdPA-Cd).

**FIGURE 1:**
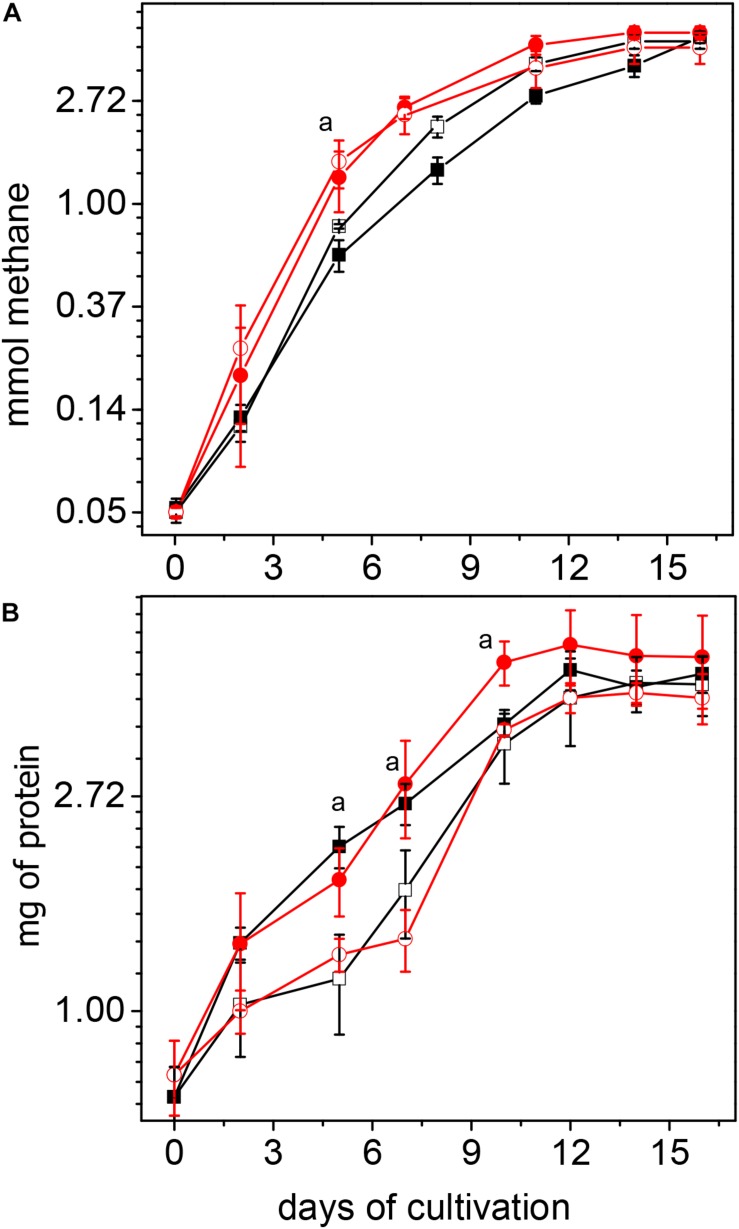
Growth of *Methanosarcina acetivorans* cultured with acetate. Methane production **(A)** and protein synthesis **(B)** were determined in control cell cultures (□); control cells exposed for first time to Cd^2+^ (■); cultures with CdPA cells (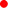) and CdPA cells cultures where Cd^2+^ was omitted for first time (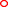). Rates of methane production determined by considering the linear part of the plot (up to day 5) in mmole x day^–1^ were: Cnt cells: 0.15 ± 0.03; Cnt + Cd: 0.13 ± 0.02; CdPA: 0.25 ± 0.05 and CdPA-Cd: 0.29 ± 0.06. Values shown are the mean ± SD of 3 independent experiments. ^a^ *P* < 0.05 in methane synthesis at day 5 between CdPA vs. Cnt and Cnt + Cd; as well as in protein content at day 5 and 7 between CdPA vs. Cnt and CdPA-Cd and at day 10 vs. cnt, cnt + Cd and CdPA-Cd.

Methanogenesis rates, at the exponential phase of growth (up to day 5), were higher in acetate-grown cultures of CdPA and CdPA-Cd vs. Cnt cells, whereas the protein contents were higher in CdPA and Cnt + Cd cells (Figure 1). ATP contents ranged 3.7–4.7 nmol (mg protein)^–1^ among the different cell culture conditions. ATP/ADP ratios were 1.2 for Cnt and Cnt + Cd cells and 1.6 for CdPA and CdPA-Cd cells. These data suggested that Cd^2+^ chronic exposure did not affect energy metabolism. In methanol-grown cells, no differences in methane production or cellular protein content were found among the four different cells used in this study (data not shown). Since no differences in phenotype developed in presence of methanol, and acetate is *M. acetivorans* “natural” substrate, no further experimentation was carried out in methanol-grown cells.

Chronic Cd^2+^ exposure in acetate-grown CdPA cells increased both the content of thiol-containing metabolites (1.25 *vs.* 0.48 μmoles thiols in Cnt + Cd cells; *n* = 2) and the Cd^2+^ removal capacity (2.56 ± 0.86 *vs.* 1.38 ± 0.25 μmoles Cd^2+^ in Cnt + Cd cells; *n* = 3; X ± SD; *P* < 0.005). Thiol/Cd^2+^ ratios were 0.49 and 0.35 for CdPA and Cnt + Cd, respectively. Acetate-grown CdPA-Cd cells still contained intracellular Cd^2+^ after one culture in the absence of metal (0.78 ± 0.01 μmoles Cd^2+^; X ± SD; *n* = 3). Thiol-metabolites content (0.20 μmoles thiols; *n* = 2) and thiol/Cd^2+^ ratio (0.25) were lower in CdPA-Cd cells than in Cnt + Cd cells.

### Phosphate Metabolism Genes in Archaea and Effect of Cd^2+^ Stress on Polyp Content

Low thiol/Cd^2+^ ratios in CdPA cells suggested that other chelating molecules, such as polyP, could be involved in binding the accumulated metal ions. It has been reported that PPX and Pi transporters may be involved in Cu^2+^ resistance in the archaeon *Metallosphaera sedula* (Rivero et al., 2018). These observations led us to survey genes coding for proteins involved in polyP metabolism (*ppx*, *ppk* and phosphate transporters). Methanogens and other archaea genomes available in the KEGG data base were inspected^1^.

Polyphosphate kinase or polyP/ATP NAD^+^ kinase, and PPX or Ppx/GppA phosphatase or NAD^+^-binding component fused to domain related to PPX genes were found in *Methanosarcina sp.*, other methanogens and other archaea. Some exceptions are *Methanohalobium* and *Methanosalsum* (Methanosarcinales) and *Thermoplasma* (Thermoplasmata). Regarding the phosphate transporter, most of the genomes analyzed showed genes encoding the *pst* (phosphate specific transport) *operon*, the ABC type phosphate transporter or both (Supplementary Table S1). The widespread localization of these genes among Archaea suggested an essential role for polyP in cell homeostasis, and potentially as mechanism of resistance by binding and accumulating heavy metals. Therefore, the biochemical study of polyP metabolism appears crucial to understand its physiological role in enhanced heavy metal accumulation phenotypes.

**FIGURE 2:**
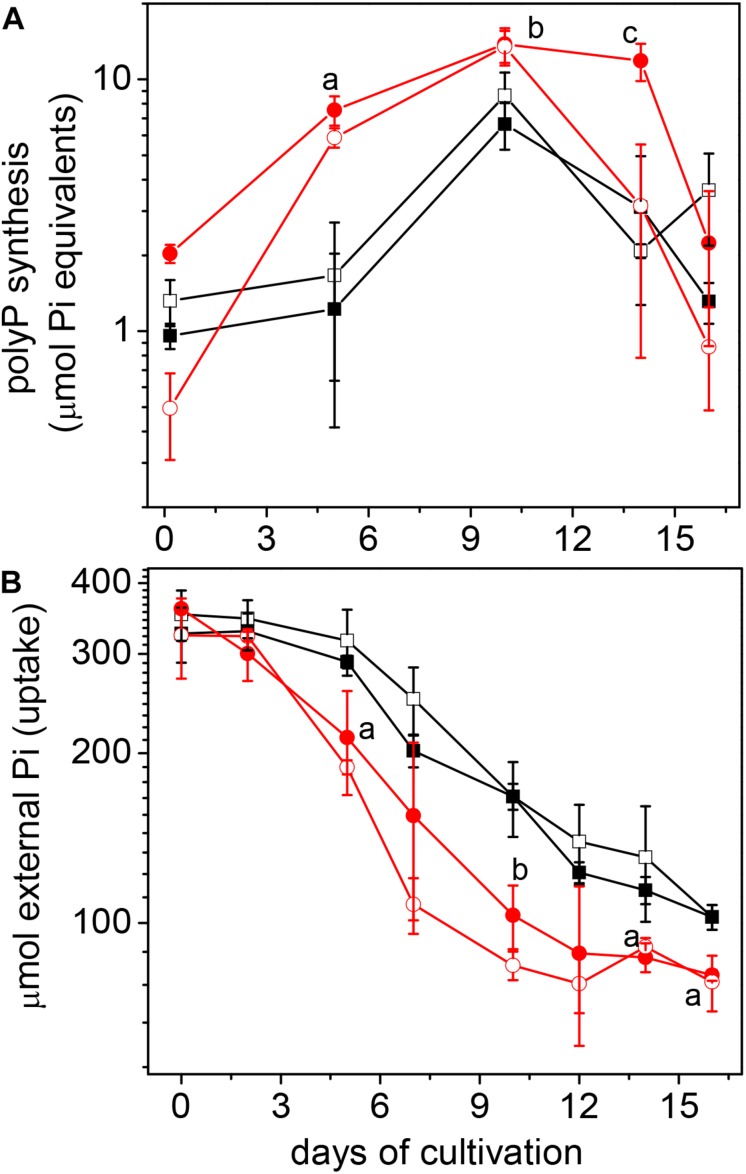
Phosphate metabolism in *Methanosarcina acetivorans* cultured in acetate. PolyP synthesis **(A)** and phosphate uptake **(B)** were determined in Cnt (□), Cnt + Cd (■), CdPA (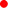) and CdPA-Cd (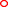) cells. Values shown are the mean ± SD of 3 independent experiments. PolyP synthesis: ^a^*P* < 0.01 and ^b^*P* < 0.05 CdPA vs. Cnt and Cnt + Cd; ^c^*P* < 0.01 CdPA vs. Cnt, Cnt + Cd and CdPA-Cd. Pi uptake: ^a^*P* < 0.05; ^b^*P* < 0.01 CdPA vs. Cnt and Cnt + Cd.

The synthesis of polyP, driven by active Pi uptake, was stimulated by Cd^2+^ at the exponential growth phase (Figure 2A). Extracellular Pi was in excess, since approximately 1.6 mM Pi remained in the medium at the end of the growth curve (Figure 2B). The high polyP/Cd_removal_ ratios (>2; Table 1), *plus* the thiol-molecule content, sufficed to completely inactivate intracellular accumulated Cd^2+^. Indeed, elemental analysis of the acetate-grown CdPA cells by high-angle annular dark-field imaging scanning transmission electron microscope (HAADFSTEM) showed numerous electrodense granules (acidocalcisomes; Supplementary Figure S1A), in which Cd, P, S, and Ca were all detected (Supplementary Figures S1B,C). On the contrary, acidocalcisomes were scarce in Cnt cells (Supplementary Figure S1D).

**TABLE 1:** PolyP content and cadmium removal in *Methanosarcina acetivorans.*

	**Acetate-grown cells**			**Methanol-grown cells**		
		
**Growth condition**	**polyP**	**Cd^2+^ removal**	**polyP/Cd^2+^ ratio**	**polyP**	**Cd^2+^ removal**	**polyP/Cd^2+^ ratio**
				
	**μmol**			**μmol**		
Cnt	2.3 ± 0.1	—	—	2.5 ± 1		—
Cnt + Cd	3.6 ± 1.4	1.4 ± 0.2	2.7	3.6	0.99 ± 0.3	2.6
CdPA	12 ± 2^a^	2.6 ± 0.8^c^	4.6	7.4	2.8 ± 0.7	2.9
CdPA-Cd	3.4 ± 2^b^	0.78 ± 0.01	4.3	Nd	Nd	Nd

*Values shown were determined at day 14 of the growth curve and are the mean ± SD of 3 independent experiments. Student *t*-test for non-paired samples, ^*a*^*P* < 0.01 *vs.* control. ^*b*^*P* < 0.05 *vs.* CdPA. ^*c*^*P* < 0.05 vs. Cnt + Cd. Nd; not determined. Cellular protein content *per* culture was 5 and 10 mg, for acetate- and methanol-grown cells, respectively (Lira-Silva et al., 2012).*

When cells were cultured under limiting extracellular 0.05 mM Pi, low polyP and higher content of thiol molecules in all growth conditions were detected (Supplementary Figure S2). The cell Cd^2+^ removal capacity could not be reliably determined due to an extensive cell agglomeration, although no Cd^2+^ was detected in the supernatant (data not shown).

### Transcriptomic and Kinetic Analyses of the Enzymes Involved in PolyP Metabolism

**FIGURE 3:**
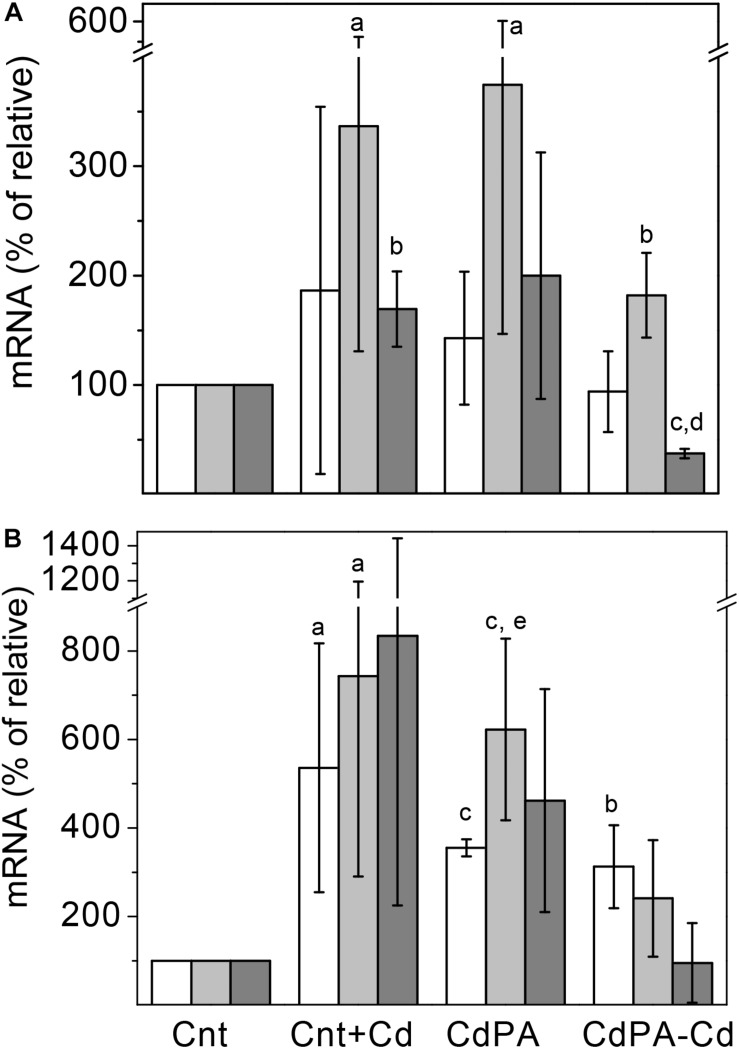
Relative transcript contents of the *ppx and ppk* genes in *Methanosarcina acetivorans.* Transcript contents of the *ppk* (white bars), *ppx* (light gray bars) and *pstA* (gray bars) genes were determined in Cnt + Cd, CdPA and CdP-Cd cells and compared to control cells (Cnt), after 7 **(A)** and 14 **(B)** days of culture. Data shown are the mean ± SD of three independent experiments carried out by triplicate. ^a^*P* < 0.05, ^b^*P* < 0.01, ^c^*P* < 0.001 vs. Cnt cells, ^d^*P* < 0.01 vs. Cnt + Cd and CdPA, ^e^*P* < 0.05 vs. CdPA-Cd.

To unveil whether the synthesis/degradation of polyP is modified by Cd^2+^ in *M. acetivorans*, transcript levels or/and activities of the Pi transporter subunit A (*pstA*), PPK (*ppk*) and PPX (*ppx*) were determined. Transcript levels of the *ppk* gene did not vary among the different cell types at day 7 of culture but increased at day 14. In turn, *ppx* gene transcript levels increased in Cnt + Cd, CdPA and CdPA-Cd cells, with respect to Cnt cells, after 7 or 14 days of culture (Figure 3). CdPA and Cnt + Cd transcript contents of the *pstA* gene increased at both days 7 and 14 but decreased by 50% in the CdPA-Cd cells at day 7 (Figure 3). PPK and PPX activities in CdPA cells, at day 7, increased as compared to Cnt cells (Table 2). At day 14 of growth, PPK activity was negligible in all cell types whereas PPX activities increased, but only significantly in CdPA-Cd cells (Table 2).

**TABLE 2:** Polyphosphate kinase and PPX activities in *Methanosarcina acetivorans.*

	**7 days of culture**		**14 days of culture**	
		
	**PPK**	**PPX**	**PPK**	**PPX**
Cnt	19 ± 6 (8)	<1 (8)	<1 (3)	80 ± 30 (4)
Cnt + Cd^2+^	20 ± 8 (6)	7 ± 6 (6)	<1 (3)	45 ± 27 (3)
CdPA	58 ± 20^a,b^ (3)	10 ± 9 (3)	7 ± 3 (4)	68 ± 24 (4)
CdPA-Cd^2+^	40 ± 18 (3)	<1 (3)	<1 (3)	174 ± 53^c,d,e^ (3)

*Activities were determined in cytosolic fractions from the indicated types of cells, all grown in acetate as carbon source. The detection limit of the assay is ∼ 1 nmol (min^–1^ x mg protein)^–1^. Values are the mean ± SD. Number of independent preparations assayed is indicated in parentheses. ^*a*^*P* < 0.01 *vs.* Cnt cells, ^*b*^*P* < 0.01 *vs*. Cnt + Cd^2+^ cells, ^*c*^*P* < 0.05 *vs.* Cnt cells, ^*d*^*P* < 0.025 *vs.* CdPA cells, ^*e*^*P* < 0.025 *vs*. Cnt + Cd^2+^ cells.*

Heterologous transcription of the *ppk* and *ppx* genes resulted in 2187 and 1632 bp products, respectively, which were the expected sizes according to the NCBI-Gene (ID 1471973 for *ppk* and ID 1471975 for *ppx*) *loci* (Supplementary Figure S3A). In turn, cloning, heterologous overexpression and purification resulted in 81 and 61 kDa proteins for the recombinant Ma-PPK and Ma-PPX, respectively (Supplementary Figure S3B). Both enzymes showed activity using commercial trimethylsilyl polyP as polyP source in the presence of 120 mM K^+^, the physiological intracellular concentration in *M. acetivorans* (Santiago-Martínez et al., 2016). Both enzymes displayed Michaelis-Menten type kinetics (Supplementary Figures S4A–C); 120 mM K^+^ decreased the *Vmax* value of Ma-PPK and increased that of Ma-PPX, whereas their *Km* values remained unchanged (Supplementary Table S2). Also, K^+^ induced a change in Ma-PPX kinetics, from hyperbolic (Michaelis-Menten) to sigmoidal (Hill) behavior (Supplementary Figure S4D) with a Hill value ≥2 (Supplementary Figure S4D, insert). To determine the specific regulating effect of K^+^, other monovalent cations were also tested; 120 mM K^+^ or Li^+^, 50 mM Na^+^ and 10 mM NH_4_^+^ or Rb^+^, indeed inhibited the PPK activity by more than 90% (*n* = 2). In turn, PPX activity increased by 2.5 times with 120 mM K^+^, Li^+^ or Na^+^ with respect to activity in absence of monovalent cations (*n* = 2).

**FIGURE 4:**
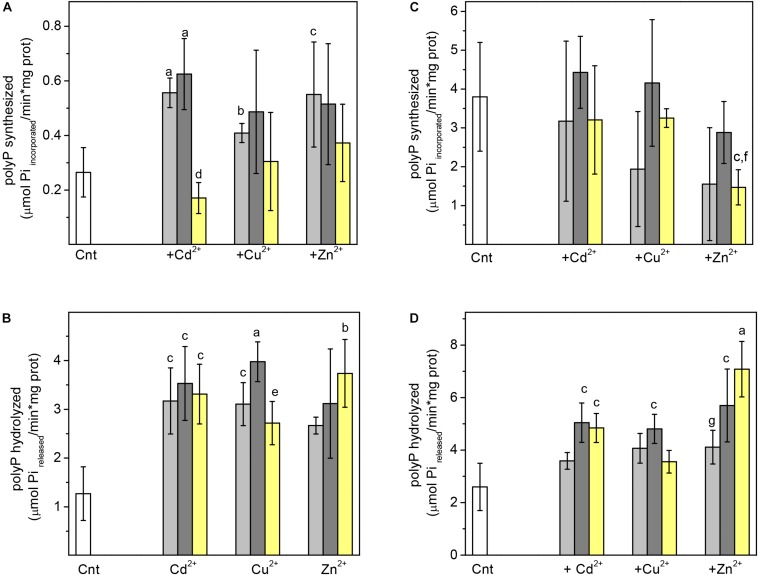
Effect of heavy metal on the activity of recombinant Ma-PPK and Ma-PPX Activities of PPK and PPX in presence **(A,B)** or absence of 120 mM K^+^ **(C,D)**, respectively, were determined in absence (white bar) or presence of 10 (light gray bar), 25 (dark gray bar) or 50 μM (yellow bar) of the indicated heavy metal. The concentration of substrates was for PPK **(A,C)** 2.5 mM ATP and for PPX **(B,D)** 5 mg Trimethylsilyl polyP/mL, respectively. Student *t*-test for non-paired samples, ^a^*P* < 0.01; ^b^*P* < 0.025; ^c^*P* < 0.05 *vs.* Cnt without heavy metal; ^d^*P* < 0.01 *vs*. 10 and 25 μM Cd^2+^; ^e^*P* < 0.05 *vs*. 25 μM Cu^2+^; ^f^*P* < 0.05 *vs*. 25 μM Zn^2+^; ^g^*P* < 0.025 *vs*. 50 μM Zn^2+^.

**FIGURE 5:**
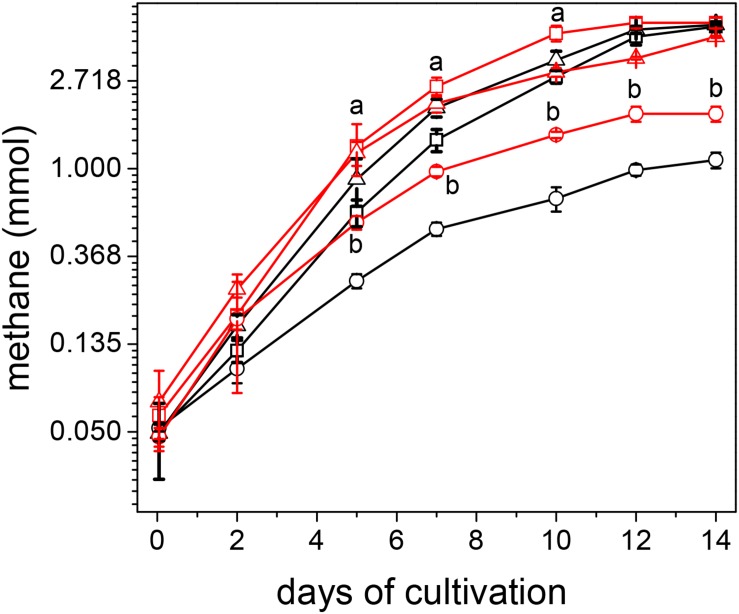
Growth of *Methanosarcina acetivorans* cultured with Cu^2+^ or Zn^2+^. Methane production was determined in control cell cultures in absence (□) or in presence of Cu^2+^ (◦) or Zn^2+^ (△) as well as in CdPA cells in absence (■) or in presence of Cu^2+^ (•) or Zn^2+^ (▲). Concentration was 100 μM for Cu^2+^ and Zn^2+^. It is noted that when CdPA cells were cultured in presence of Cu^2+^ or Zn^2+^, the 50 μM Cd^2+^ was not added for first time. Values shown are the mean ± SD of 3 independent experiments. ^a^*P* < 0.01 CdPA vs. control; ^b^*P* < 0.01 CdPA *vs*. control (in presence of Cu^2+^).

Polyphosphate kinase was activated by 10 and 25 μM of Cd^2+^, Cu^2+^or Zn^2+^, whereas 50 μM did not bring about significant activity changes (Figure 4A). In turn, PPX was also 2.5–3 times activated by 10–50 μM of Cd^2+^, Cu^2+^ and Zn^2+^ (Figure 4B). The absence of K^+^ (a non-physiological condition) decreased its activity (Figures 4C,D). Interestingly, CdPA cells were more resistant to 100 μM Zn^2+^ or Cu^2+^ stress than Cnt cells (Figure 5), suggesting that enhanced polyP metabolism also served for other divalent cations inactivation.

Recombinant enzyme activities were also determined with an enriched-fraction of native polyP isolated from *M. acetivorans*. Ma-PPK showed detectable activity using 30 μg native polyP/mL as substrate, with a *Vmax* of 0.06 ± 0.02 μmol polyP_synthesized_ (min × mg prot)^–1^, (X ± SD; *n* = 5), whereas Ma-PPX required 0.3–1.2 mg native polyP/mL, showing a *Vmax* of 0.3 ± 0.2 μmol polyP_hydrolyzed_ (min × mg prot)^–1^ (*n* = 4).

### Biofilm Synthesis

Acetate-grown Cnt cells were able to synthesize biofilm, but a single exposure to >250 μM Cd^2+^ was toxic and diminished by 75% their biofilm synthesis capacity. In contrast, the CdPA cells synthesized eight times more biofilm with respect to Cnt cells after 14 days (Supplementary Figure S5).

### Genome Sequencing

The genomes of *M. acetivorans* Cnt and CdPA cells were successfully assembled into high-quality drafts. The raw data comprised a total of 51,501,938 and 50,029,628 reads that were assembled into 382 and 348 contigs with N50 of 28,555 and 32,300 for Cnt and CdPA cells, respectively. This complete genome project has been deposited in GenBank and the data are available through BioProject accession number PRJNA477340 and biosample accession numbers are SAMN09467189 and SAMN09467190. Quality of the genome assembly was assessed with QUAST (Gurevich et al., 2013). Total length of the contigs were ordered and compared with *M. acetivorans* C2A (NC_003552.1, May 2017) reference genome (Galagan et al., 2002) using Mauve version 2.0 (Rissman et al., 2009); they were very similar, with no gaps or inversions observed. Genome sequence and annotation results showed that both genomes were identical, with respect to the number of the genes. Further genome analysis was carried out to identify genes participating in thiol-metabolites or polyP synthesis, antioxidant defense and gluconeogenic metabolism. This was an initial attempt to find the source of phenotypic differences between CdPA and Cnt strains. From the 51 analyzed genes, and in agreement with the whole sequence genome, no differences were found in CdPA cells compared to the reference genome. An unexpected finding was that 11 genes in the CdPA genome, encoding proteins with no putative roles assigned yet, showed changes in identity, length and *E*-value with respect to the *M. acetivorans* genome used as a reference (Supplementary Table S3).

## Discussion

### Generalities

In a wide variety of organisms such as the protist *Euglena gracilis* (Avilés et al., 2003), algae, plants (Küpper et al., 2002; Ranjard et al., 2008; Meyer et al., 2016) and *M. acetivorans* (Jasso-Chávez et al., 2015), the pre-adaptation to low doses of stressors such as heavy metals or O_2_ triggers the development of resistance mechanisms toward higher stressor concentrations. In the present work, it was shown that under chronic Cd^2+^ exposure increased rates of methane synthesis were attained, in agreement with previous reports where acetate-grown cells but not methanol-grown cells increase their methane production rates in the presence of Cd^2+^ (Lira-Silva et al., 2012, 2013). In addition, the protein content was higher in CdPA and Cnt + Cd cells with respect to Cnt cells. This suggested that indeed Cd^2+^ may activate transcription factors that promotes cell duplication.

Genes encoding proteins involved in polyP metabolism are widespread in Archaea (Supplementary Table S1). It has been suggested that under nutritional stress, polyP provides Pi for ATP production and metabolism, thus preserving cell viability (Brown and Kornberg, 2008). Furthermore, it has also been proposed that in the archaea *Pyrococcus horikoshii, M. acetivorans, Archaeoglobus* sp. and *Metallosphaera sedula* as well as in the proteobacteria *Acidithiobacillus ferrooxidans*, enhanced polyP and thiol-metabolite syntheses are two of the main mechanisms used to deal with the toxicity of Cu^2+^ and Cd^2+^ (Alvarez and Jerez, 2004; Lira-Silva et al., 2013; Toso et al., 2016; Rivero et al., 2018). In the present work, it was determined that CdPA cells cultured once without Cd^2+^ still contained intracellular Cd^2+^, probably because the metal ion was tightly bound by thiol-containing molecules (Lira-Silva et al., 2012) and polyP.

It was previously determined that *M. acetivorans* cells contained polyP granules (acidocalcisomes) even when cells were grown without Cd^2+^ and phosphate (Lira-Silva et al., 2012). The present study demonstrated that polyP granules increased under persistent Cd^2+^ exposure. These data suggested that polyP is not only synthesized for Pi storage, but also as a protective mechanism to bind and neutralize divalent cations at toxic concentrations.

Noteworthy, the polyP contents were much higher than those of the intracellular thiol-containing molecules at 5 mM external Pi. On the contrary, when phosphate was limiting (0.05 mM), thiol-containing molecules prevailed over polyP synthesis. These data suggested that the mechanisms raised by *M. acetivorans* against Cd^2+^ depend on external sources of sulfur and phosphorus.

### Regulation of PolyP Metabolism

The roughly constant *pstA* transcript level, except for CdPA-Cd cells at day 7 of growth, correlated with an active external Pi uptake. In addition, an increased Pi transport into cells also correlated with an active polyP synthesis in the presence of Cd^2+^. In *M. acetivorans* the *ppk* and *ppx* genes are in the same operon, as in *E. coli* (Akiyama et al., 1993). Cd^2+^ activated the transcription of both, *ppk* and *ppx* genes in Cnt + Cd and CdPA cells. However, no correlation was found between transcript contents and enzymatic activities, i.e., PPX activity at day 7 and PPX activity at day 14 were low or negligible, whereas their transcript levels were significant. These observations suggested that, besides transcriptional regulation, PPK and PPX may be also modulated at the post-translational (covalent regulation) and metabolic levels (inhibition by products) in *M acetivorans.* In turn, the higher PPX transcript level and enzyme activity in CdPA-Cd cells, with respect to control cells, was probably due to the presence of remaining internal Cd^2+^

It is well known that many enzymes are regulated by metal ion binding into the catalytic site, and they become efficient and selective promoters of catalysis and/or stability (Gohara and Di Cera, 2016). In the present work, Zn^2+^, Cd^2+^, and Cu^2+^, as well as K^+^ displayed a regulatory role of Ma-PPK and Ma-PPX activities. It has been suggested that K^+^ may serve to position and activate the phosphoryl transfer process (Jurica et al., 1998). Similarly, PPX from *Chlorobium tepidum and E. coli* are activated by 20 and 175 mM K^+^, respectively (Proudfoot et al., 2004; Albi and Serrano, 2014). In turn, PPK from *Sulfolobus acidocaldarius* is activated by 2 mM K^+^ or Mg^2+^ (Skórko et al., 1989); no inhibitory effect of K^+^ on PPK has been reported.

Heavy metal cations stimulated both PPK and PPX activities in *M. acetivorans*. Similarly, PPX from *S. cerevisiae* and *Corynebacterium glutamicum* are activated by 0.05 Co^2+^ and 2 mM Zn^2+^, respectively (Andreeva et al., 2004; Lindner et al., 2007). When comparing PPX with the superfamily of metal-dependent phosphohydrolases (Aravind and Koonin, 1998), BLASTp analysis identified a “HD” (His-Asp) motif in PPX at amino acids 345–468 (Aravind and Koonin, 1998). Therefore, this region may be involved in the divalent cation activation of Ma-PPX. In contrast, some heavy metals are inhibitors of *E. coli* and *C. glutamicum* PPK (Ahn and Kornberg, 1990; Lindner et al., 2009). These differences in the effect of divalent cations are consistent with a low amino acid identity among different species. Alignment of the *M. acetivorans* PPK (gene ID 1471973) with protein sequences of other microorganisms revealed 35–44% identity with high query cover of 94% (Supplementary Figure S6A). In turn, alignment of the *M. acetivorans* PPX (gene ID 1471975) showed 26–33% identity and wide range 54–94% query cover (Supplementary Figure S6B).

Physiological (K^+^, Na^+^, and NH_4_^+^) and non- physiological (Li^+^ and Rb^+^) monovalent cations affected the activity of both Ma-PPK and Ma-PPX, suggesting a specific binding site for K^+^/Na^+^. Whether the binding sites of divalent cations are different from those for K^+^, or Cd^2+^ modifies the intracellular concentration of K^+^ to further regulate PPK and or/PPK remains to be determined.

Ma-PPK showed a *Km*_ATP_ = 1.4 mM whereas the intracellular ATP concentration ranged 5–6.5 mM (3.5–5 times the *Km* value), suggesting that in *M. acetivorans* this enzyme does not display its maximal catalytic potential; in contrast, the high intracellular polyP levels (Figure 2A) and *Km_polyP_* = 1.2 mg/mL indicated that the Ma-PPX substrate was saturating. Moreover, Ma-PPK showed a lower catalytic efficiency with respect to Ma-PPX (i.e., *Vmax/Km* of 0.7 *vs.* 3, respectively; see Supplementary Table S2). Thus, these data suggested that Ma-PPK may have a higher controlling role on polyP homeostasis because it is slower and less efficient than Ma-PPX.

The CdPA phenotype was reverted once Cd^2+^ was omitted from the culture media. As cell biomass (see Figure 1) and levels of Cd^2+^-binding molecules (Figures 2A and Supplementary Figure S3) were lower in CdPA-Cd cells than in CdPA cells, it is suggested that these cell functions and in general the CdPA phenotype are regulated by the presence of the metal and not determined by point mutations, at least in genes coding for proteins involved in the metabolism of carbohydrates, sulfur or phosphate. Further cultivation of CdPA cells with 100 μM Cu^2+^ or Zn^2+^ produced more biomass than that determined for Cnt cells; this observation deserves further analysis.

## Conclusion

Persistent exposure to Cd^2+^ activated polyP metabolism. The mechanisms here described may provide insights on how phosphate metabolism in *M. acetivorans* and other archaea is regulated and therefore, the present study proposes the use of these organisms as a potential tool for the removal of metals and phosphate in polluted waters with the associated benefit of high biogas yields.

## Data Availability Statement

The datasets generated for this study can be found in the complete genome project has been deposited in GenBank, and the data are available through BioProject accession number PRJNA477340 and biosamples accession numbers are SAMN09467189 and SAMN09467190.

## Author Contributions

RJ-C, EL-S, and RM-S: conceptualization and methodology. RJ-C and RM-S: resources. VL-S, DM-M, and FP-V: data curation. EL-S, KG-S, AV-S, and AZ-R: investigation. R-JC, EM, and RM-S: formal analysis. RJ-C: original draft. RJ-C, MT-M, and RM-S: writing-review and editing.

## Conflict of Interest

VL-S and DM-M were employed by company Winter Genomics. The remaining authors declare that the research was conducted in the absence of any commercial or financial relationships that could be construed as a potential conflict of interest.

## Footnotes

1 http://www.genome.jp/kegg/
