# Supplementary material for: Marine Archaeon Methanosarcina acetivorans Enhances Polyphosphate Metabolism Under Persistent Cadmium Stress
Source: Front Microbiol. 2019 Oct 24;10:2432. doi: 10.3389/fmicb.2019.02432 (PMC6821655; doi:10.3389/fmicb.2019.02432)
Supplement: Supplementary file 1 [file Table_1.docx]

Supplementary Table 1 **Genes encoding for PPK, PPX and phosphate transporter PstA in methanogens and other archaea**

| **Organism** | *ppk* | *ppx* | Pi permease protein A (*pstA*) |
| --- | --- | --- | --- |
| *Methanocaldococcus* | x | Mn-dependent inorganic Pyrophosphatase | √ |
| [*Methanotorris*](http://www.genome.jp/kegg-bin/show_organism?category=Methanotorris) | (poly(P)/ATP NAD kinase | x | √ |
| *Methanococcus* | (poly(P)/ATP NAD kinase | x | √ |
| *Methanothermococcus* | (poly(P)/ATP NAD kinase | Ppx/GppAphosphatase | √ |
| *Methanosarcina* | √ | √ | √ |
| *Methanococcoides* | x | NAD-binding component fused to domain related to PPX | √ |
| *Methanohalophilus* | √ | Ppx/GppA phosphatase | √ |
| *Methanohalobium* | x | x | √ |
| *Methanosalsum* | x | x | √ |
| *Methanolobus* | √ | √ | √ |
| *Methanomethylovorans* | √ | √ | √ |
| *Methanosaeta* | putative (poly(P)/ATP NAD kinase | x | √ |
| *Methanospirillum* | √ | √ | Pi transporter,  PiT family |
| *Methanocorpusculum* | √ | Ppx/GppA phosphatase | √ |
| *Methanoculleus* | putative (poly(P)/ATP NAD kinase | x | putative ABC transporter permease protein yqgH |
| *Methanolacinia* | √ | Ppx/GppA phosphatase | √ |
| *Methanoregula* | √ | √ | √ |
| *Methanosphaerula* | √ | √ | √ |
| *Methanocella* | probable (poly(P)/ATP NAD kinase | Exopolyphosphatase-related protein | √ |
| *Methanothermobacter* | probable (poly(P)/ATP NAD kinase | x | √ |
| *Methanosphaera* | √ | x | √ |
| *Methanobrevibacter* | √ | √ | √ |
| *Methanobacterium* | √ | √ | √ |
| *Methanothermus* | x | √ | Pi transporter,  PiT family |
| *Methanopyrus* | x | NAD-binding component fused to domain related to PPX | √ |
| *Methanomassiliicoccus* | √ | Ppx/GppAphosphatase | phosphate ABC transporter |
| *Archaeoglobus* | (poly(P)/ATP NAD kinase | √ | √ |
| *Thermoplasma* | x | x | Pi transporter related protein |
| *Pyrococcus* | poly(P)/ATP NAD kinase | x | √ |
| *Thermococcus* | poly(P)/ATP NAD kinase | NAD-binding component fused to domain related to PPX | √ |
| *Sulfolobus* | √ | √ | √ |
| *Metallosphaera* | x | Ppx/GppA phosphatase | general substrate transporter |
| *Pyrobaculum* | x | PPX-related protein | ABC transporter, permease |
| *Thermoproteus* | poly(P)/ATP NAD kinase | PPX-related protein | Pi transporter related protein |

Genes are indicated as present (√) or absent (x). Ppx/GppAphosphatase: PPX/ guanosine-5'-triphosphate, 3'-diphosphate pyrophosphatase. Analysis was carried out with the KEGG (KEGG; http://www.genome.jp) reported genes. Blue lines denoted archaea bearing the three evaluated genes. In this comparison *Sulfolobus* was the only archaea belonging to Crenarchaeota containing the three evaluated genes.
