# Supplementary material for: Marine Archaeon Methanosarcina acetivorans Enhances Polyphosphate Metabolism Under Persistent Cadmium Stress
Source: Front Microbiol. 2019 Oct 24;10:2432. doi: 10.3389/fmicb.2019.02432 (PMC6821655; doi:10.3389/fmicb.2019.02432)
Supplement: Supplementary file 2 [file Table_2.docx]

Supplementary Table 2 **Kinetic parameters of recombinant Ma-PPK and Ma-PPX**

|  | *Vmax* | | *Km* | | *Vmax/Km* | |
| --- | --- | --- | --- | --- | --- | --- |
|  | - K^+^ | + K^+^ | - K^+^ | + K^+^ | - K^+^ | + K^+^ |
| PPK | 3.7 ± 0.74 (4) | 1 ± 0.4 (4) | 0.8 ± 0.35 mM ATP | 1.4 ± 0.5  mM ATP | 4.6 | 0.71 |
| PPX | 7.5 ± 2.6 (4) | 19 ± 4 (4) | 0.7 ± 0.4  mg Trimethylsilyl  polyP/mL | 1.2 ± 0.3  mg Trimethylsilyl  polyP/mL  n=2.8 ± 1 (Hill) | 2 | 3 |

Kinetic parameters were determined in the absence (-K^+^) or presence of 120 mM KCl (+K^+^). Activity units are, µmol polyP _synthesized_ or polyP _hydrolyzed_ (min x mg protein)^-1^ for Ma-PPK and Ma-PPX, respectively. For the PPX *Vmax/Km* value, *Vmax* values were transformed to 1.4 ± 0.5 and 3.5 ± 0.7 mg _polyP_ (min x mg protein)^-1^. Values are the mean ± SD of 4 different preparations.
