# Supplementary material for: Marine Archaeon Methanosarcina acetivorans Enhances Polyphosphate Metabolism Under Persistent Cadmium Stress
Source: Front Microbiol. 2019 Oct 24;10:2432. doi: 10.3389/fmicb.2019.02432 (PMC6821655; doi:10.3389/fmicb.2019.02432)
Supplement: Supplementary file 3 [file Table_3.docx]

Supplementary Table 3. Genes identified with differences in identity, large E value or Bitscore at nucleotide level.

|  | Former gene ID | Current gene ID | Putative function | Position (bp) |
| --- | --- | --- | --- | --- |
| 1 | MA2943 | MA_RS15410 | Predicted protein | 3660001 to 3810000 |
| 2 | MA_1787 | MA_RS09285 | Predicted protein | 2190001 to 2340000 |
| 3 | MA_1242 | MA_RS06450 | Predicted protein | 1470001 to 1620000 |
| 4 |  | MA_RS25385 | IS1 family transposase | 2194378 to 2195473 |
| 5 |  | MA_RS16365 | Predicted protein | 3888709 to 3888897 |
| 6 |  | MA_RS26000 | Predicted protein | 4586320 to4586574 |
| 7 |  | MA_RS25120 | IS1-like element ISMac25 family transposase | 1240110 to 1241205 |
| 8 | MA_2800 | MA_RS25695 | IS1182 family transposase ISMac20 | 3493235 to 3494830 |
| 9 | MA_1764 | MA_RS24475 | PKD domain-containing protein | 2152449 to 2155169 |
| 10 | MA_2770 | MA_RS25665 | IS1182-like element ISMac20 family transposase | 3453640 to 3455235 |
| 11 | MA0385 | MA_RS02025 | Cobalt chelatase subunit CobN | 458726 to 463399 |
