# Supplementary material for: Marine Archaeon Methanosarcina acetivorans Enhances Polyphosphate Metabolism Under Persistent Cadmium Stress
Source: Front Microbiol. 2019 Oct 24;10:2432. doi: 10.3389/fmicb.2019.02432 (PMC6821655; doi:10.3389/fmicb.2019.02432)
Supplement: Supplementary file 4 [file Table_4.docx]

Supplementary Table 4 **Primers sequences used for transcript levels determinations**.

| gene | Gen ID (NCBI) | gene | Product size (bp) | Sequence |
| --- | --- | --- | --- | --- |
| MA0081 | 1471973 | *ppk* | 140 | Fw 5´ -GAATTCCTGAGCAACCCATGC -3´ |
|  |  |  |  | Rw 5´ -GGATCCTCAGCTCTTTTCCGT-3´ |
| MA0083 | 1471975 | *ppx* | 123 | Fw 5´ -GCGAATTCATGGAACCCGAGAAA-3´ |
|  |  |  |  | Rw 5´ -GGATCCTCATTCCCGAGGGAC-3´ |
| MA0889 | 1472781 | *pstaA* | 150 | Fw 5´ -ATATGCAGGAGAAAACACGCA-3´ |
|  |  |  |  | Rw 5´ -GTGAGGGAAGCCGACAGTAT-3´ |
| MA1018 | 1472908 | *gapd* | 115 | Fw 5´ -GCTATCCACCAGGAGTCCAAT-3´ |
|  |  |  |  | Rw 5´ -GCCCAATAGCCTTGTTTGTT-3´ |
