# Supplementary material for: Marine Archaeon Methanosarcina acetivorans Enhances Polyphosphate Metabolism Under Persistent Cadmium Stress
Source: Front Microbiol. 2019 Oct 24;10:2432. doi: 10.3389/fmicb.2019.02432 (PMC6821655; doi:10.3389/fmicb.2019.02432)
Supplement: Supplementary file 5 [file Table_5.docx]

Supplementary Table 5. **CT values for *gapd* transcript used as housekeeping gene in *M. acetivorans***.

| Growth condition | CT value (min) |
| --- | --- |
| Cnt | 22.4 ±1.8 |
| Cnt+Cd | 21.5 ± 1.2 |
| CdPA | 25.5 ± 2.6 |
| CdPA-Cd | 20.3 ± 0.3 |

Values shown are the mean ± SD of 4 (Cnt+Cd and CdPA-Cd) or 5 (Cnt and CdPA) cDNA preparations made by triplicate.
