# Supplementary material for: Marine Archaeon Methanosarcina acetivorans Enhances Polyphosphate Metabolism Under Persistent Cadmium Stress
Source: Front Microbiol. 2019 Oct 24;10:2432. doi: 10.3389/fmicb.2019.02432 (PMC6821655; doi:10.3389/fmicb.2019.02432)
Supplement: Supplementary file 6 [file Table_6.docx]

Text S1

**Methods**

***S1. Heavy metal content, biofilm synthesis and ultrastructure analysis***

An analytical procedure for determining heavy metal content was performed after 14 days of culture by harvesting and washing the cells with TME-Na buffer solution. This procedure was followed by H_2_SO_4_ + HNO_3_ (1:3) digestion (5 mg protein/mL) for 4 h at 100°C. Cd, Zn, and Cu were quantitated by atomic absorption spectrophotometry in the clarified digested samples (Lira-Silva *et al*., 2012).

Biofilm synthesis was detected after 12 days of culturing acetate-grown Cnt, CdPA, Cnt+Cd, and CdPA-Cd cells with the indicated Cd^2+^ concentrations. Initial cell inoculum, equivalent to 50-75 µg protein, were placed on 96-well polystyrene plates. Biofilm formation was identified by the crystal violet method (Lira-Silva *et al*., 2013). For ultrastructure analysis, acetate-grown cells with or without CdCl_2_ treated as previously reported were used (Lira-Silva *et al*., 2012). Cross-section cell samples of 1 mm^2^ were cut out with a diamond knife and embedded in 1:1 epoxy resin. To determine the subcellular location of cadmium, phosphate, and sulfur, atomic-resolution high angle annular dark-field scanning-transmission electron microscopy (HAADFSTEM) was used.

***S2. Transcript levels determination***

Total RNAs were extracted from 7 and 14 days-old cell cultures using Quick-RNA Miniprep Kit (Zymo Research; California, USA), according to manufacturer instructions. After RNA integrity was verified by gel electrophoresis, 5 µg RNA was converted into cDNA with a RevertAid First-Strand Synthesis kit (Thermo Scientific; California, USA) and then quantified. Primers and lengths for *ppk, ppx, pstA,* and *gapd* genes products are shown in Table S4. The Maxima SYBR Green/ROX qPCR Master Mix (Thermo Scientific; California USA) was used for qRealTime-PCR analysis using 600 ng template cDNA. StepOne Real-Time PCR System (Applied Biosystems; Grand Island, NY, USA) followed protocol was: 95°C for 15 min during one cycle, followed by 40 cycles at 95°C for 15 s, 60°C for 30 s and 72°C for 30 s. The *gapd* gene was used as a house-keeping reference gene; *i.e*., its transcript content remained constant under different conditions (Santiago-Martínez *et al*., 2016). The analysis was performed in at least three independent biological samples for each experimental condition by triplicate. mRNA contents in acetate-grown control cells (without Cd^2+^; Cnt), control cells with a sole 50 µM Cd^2+^ exposure (Cnt+Cd), and CdPA cells further cultured without 50 µM Cd^2+^ for one pass (3-4 generations; CdPA-Cd cells), were determined by the 2^-ΔΔCt^ method (Livak and Schmittgen2001). .

The MA_3998 gene has been used as a house-keeping reference (Rohlin and Gunsalus, 2010; Jasso-Chávez *et al*., 2015). However, chronic Cd^2+^ stress-induced significant transcription level variation. Therefore, to find a reliable internal control gene, other gene products involved in the intermediary metabolism of *M. acetivorans* were tested*.* The tested genes were: *phosphofructokinase I* (MA_RS18605), *phosphoglucomutase* (MA_RS02360), *lactate dehydrogenase* (MA_RS24165), *glycogen synthase* (MA_RS19175), *pyruvate: phosphate dikinase* (MA_RS03195), *acetate kinase* (MA_RS18805) and *phosphotransacetylase* (MA_RS18810). No reliable transcript levels for these other genes were found (data not shown). The *gapd* gene, also previously used as a house-keeping reference, showed similar CT values under the different growth conditions and was then selected as a reference in the present work (Table S5).

***S3. Cloning and heterologous overexpression of the recombinant PPX and PPK***

DNA was isolated by using aliquots of acetate-grown cells in the early stationary phase as reported elsewhere (Jasso-Chávez *et al*., 2017). Approximately 200 μg DNA/mL were typically obtained. The acquired data were compared with the NCBI database of *M. acetivorans* C2A (AE010299.1). The cloning of MA_RS00420 and MA_RS00425 genes encoding putative PPK and PPX, respectively was accomplished with the PCR reaction. The designed primers were based on NCBI-Gene ID 1471973 for *ppk* and ID 1471975 for *ppx.* Then, *ppk* cloning primers used

were 5´GCGCTAGCAATGGAGCGGAACTTC3 as forward and 5´GCCGAATTCATCAGCTCTTTTCCGTTTTAAG3 for reverse directions. The primers contained restriction sites for Nhe I and EcoRI, respectively. The *ppx* cloning primers sequence used as forward was 5´CCGCATATGAGATGGAACCCGAGAAAATTT3´ and for reverse direction was 5´CAGGATCCGTCATTCCCGAGGGACTTT3´. Primers for *ppx* cloning contained restriction sites for Nde I and BamHI, respectively. Amplification of each gene was performed by PCR using Pfu DNA polymerase (Thermo Scientific, Waltham, Mass, USA). PCR protocols for the two genes were: one cycle for 25 s at 95°C, 40 cycles at 95°C for 30 s, annealing cycle for 30 s at 60°C, extension cycle for 90 s at 72°C and extension cycle at 72°C for 10 min. PCR products were cloned using the overexpression pJET vector (Thermo Scientific, USA) in *E. coli* Top 10 cells. *ppk* and *ppx* inserts were subcloned into pET28a vector (Novagen, Madison, WI, USA). After the sequence was verified, pET28-*ppk and* pET28-*ppx* plasmids were cloned into *E. coli* BL21DE3pLysS strain cells for enzyme overexpression.

PPK overexpression was performed by growing “PPK-*E. coli* BL21DE3pLysS” in LB media containing 50 µg kanamycin/mL, according to Saavedra *et al* (2005). PPX overexpression was achieved by the auto-induction of the PPX- *E. coli* BL21DE3pLysS cells using the ZYM-5052 medium containing 50 µg kanamycin/mL, as reported previously (Studier, 2005). Recombinant PPX was found mainly in the non-soluble fraction, and a solubilization protocol was used to increase the yield of the enzyme. The solubilized fractions were obtained by collecting the cells by centrifugation, resuspending them in the regular lysis buffer (1mM PMSF, 0.5 mM β-mercaptoethanol) containing 0.05% (w/V) Tween-20; followed by gentle stirring for 2 h at 4°C for 2 h. Afterward, a 23,489 x *g* for 20 min and 4°C centrifugation step produced the solubilized fraction. Purification of both enzymes, from the solubilized fractions, was carried out using a Cobalt TALON-metal affinity resin (Clontech, Palo Alto, California USA). The purity of the recombinant proteins was determined by densitometric analysis on 12.5% (w ⁄ v) SDS-PAGE stained with Coomassie blue.

***S4. PPK and PPX activities***

Cells cultured for 7 or 14 days were harvested by centrifugation (5000 x *g* for 10 min at 4°C), washed in TME-Na buffer and resuspended in 50 mM HEPES, 120 mM KCl and 1 mM EDTA (HKE buffer pH 7). After that, cells were disrupted by sonication in a Branson sonifier (Shelton, CT, USA) with a 2 mm tip diameter and using 60% of maximal power. Cells were subjected to three pulses of 10 s with 1 min resting in between pulses. Cell homogenates were centrifuged at 8,000 x *g* for 20 min at 4°C. The supernatant was further centrifuged at 163,000 *x g* for 45 min at 4°C; the new supernatant (cytosol-enriched fraction) was immediately used for enzyme activity determination. Activity buffers used were 50 mM HEPES, 50 mM NaCl and 10 mM MgCl_2_ at pH 7.2 (HEPES-Mg buffer), or a more “physiological buffer”: 50 mM HEPES, 50 mM NaCl, 120 mM KCl and 0.5 mM EGTA at pH 7.2 (HKE-Na buffer).

PPK activity assay mixture (0.1 mL) contained 10 mM MgCl_2_, 10 mM KH_2_PO_4,_ 15% (v/v) glycerol, 2.5 mM ATP, 0.35 mg trimethylsilyl polyP/mL, 0.2% (v/v) Triton X-100, 5 mM PEP and 1 U PYK(Mullan *et al*., 2002). Trimethylsilyl polyP (1 mg/mL) hydrolyzed by 3% PCA at 90°C for 60 min was equivalent to 5.4 ± 0.7 µmol Pi/mL (n=3). The reaction was initiated by adding the recombinant protein (1.7-4.6 µg protein) or cytosol-enriched fraction (50 µg protein) and then incubating for 60 min at 37°C under 150 rpm orbital agitation. The polyP content was determined spectrophotometrically in cell samples mixed with a dying solution (0.001% toluidine blue in 40 mM acetic acid) at 530 and 630 nm (Skorko, 1989). In comparison to the enzymatic method with PYK/LDH in which spurious NADH consumption was significant, the metachromatic method yielded more reliable results (data not shown). Ma-PPK activity was only apparent when at least 0.05 mg trimethylsilyl polyP /mL was used as core.

PPX activity was determined in a reaction buffer containing the recombinant protein (1.7-4.6 µg protein) or cytosol-enriched fraction (60-80 µg protein) and 0.2% (v/v) Tween-20. The reaction was initiated by adding 1.4 mg trimethylsilyl polyphosphate/mL. Higher polyP concentrations (*i.e.* 2 mg trimethylsilyl polyP/mL) were inhibitory (data not shown). After 15 min incubation at 37°C, the polyP hydrolysis was determined by the -Δ at 530/630 nm as described above. PPX

activity was also determined by the Pi released and detected at 870 nm by means of molybdenum blue formation (LeBel *et al*., 1978) with similar results (data not shown).

Recombinant PPK and PPX activities were also determined with endogenous polyP-enriched-fractions (30-90 and 500-1000 µg/mL, respectively). The reaction was initiated by adding the recombinant protein (1.7-4.6 µg protein) and followed for 60 min.
