# Supplementary material for: Marine Archaeon Methanosarcina acetivorans Enhances Polyphosphate Metabolism Under Persistent Cadmium Stress
Source: Front Microbiol. 2019 Oct 24;10:2432. doi: 10.3389/fmicb.2019.02432 (PMC6821655; doi:10.3389/fmicb.2019.02432)
Supplement: Supplementary file 7 [file Table_7.docx]

**Supplementary figures foot legends**

Figure S1. **Elemental analysis of acetate-grown cells by HAADF-STEM**

Electrodense zones in CdPA cells (acidocalcisomes) surrounding the cytoplasmic membrane are abundant (A) and full of Cd, P, S Mg and Ca (B and C), compared to Cnt cells (D).

Figure S2. **Intracellular metabolites in *M. acetivorans***

Contents of protein (gray), thiol-molecules (yellow) and polyP (orange) determined after 14 days of culture in either regular HS medium (5 mM orthophosphate, filled columns) or low phosphate medium (0.05 mM Pi, squared columns). Values shown are the mean of 3 independent experiments. ^a^ P<0.05; ^b^ P<0.01 *vs*. cells cultured with low Pi; ^c^ P< 0.01 *vs*. cells cultured in regular medium (5 mM Pi).

Figure S3**. Cloning, overexpression and purification of PPK and PPX of *M. acetivorans***

(A) PCR products of the *ppk* and *ppx* genes were cloned in the pET28a plasmid, digested with restriction enzymes (see the methods section) and resolved in a 1% (w/V) agarose gel (green arrows). M4 and E7 are the transformed *E. coli* clones containing the *ppk* and *ppx* inserts, respectively. Molecular markers (M) are indicated by black arrows. (B) Recombinant Ma-PPK and Ma-PPX were resolved in 10% SDS-PAGE. The molecular weights of Ma-PPK and Ma-PPX were 80.6 and 61.3 kDa, respectively. PPK and PPX purity (72% and 80%, respectively) was determined by Image J software densitometric analysis.

Figure S4. **Recombinant Ma-PPK and Ma-PPX Activity**

Kinetic parameters were obtained by non-linear regression analysis (Microcal Origin 8.0) of experimental data fitted to the Michaelis-Menten or Hill equations. PPK activity was determined in absence (A) or presence of 120 mM KCl (B) with 0.35 mg trimethylsilyl polyP/mL and indicated [ATP]. Insets show the double reciprocal plots, *Vmax*= 4.1 µmol (min x mg protein)^-1^ and *Km_ATP_*= 1 mM, in

absence of KCl; and *Vmax*= 1.1 µmol (min x mg protein)^-1^ and *Km_ATP_*= 1.7 mM when KCl was present. These kinetic parameters were similar to those obtained by non-linear regression analysis (see Table S2). PPX activity was determined at the indicated concentrations of trimethylsilyl polyP in absence (C) or presence of 120 mM KCl (D). Insets show the double reciprocal plots, *Vmax*= 8.5 µmol (min x mg protein)^-1^ and *Km_polyP_*= 1.1 mg/mL, in absence of KCl. In presence of KCl, *Vmax*= 6 µmol (min x mg protein)^-1^ and *K_0.5 polyP_*= 0.17 mg/mL. When KCl was present, the double reciprocal plot involved the reciprocal of the substrate squared values, suggesting a Hill value (n) ≥ 2, similar to that obtained by non-linear regression (see Table S2). 1 mg [trimethylsilyl polyP] hydrolyzed/mL with 3% PCA at 90°C during 60 min was equivalent to 5.4 ± 0.7 µmol Pi/mL. Data shown are the mean ± SEM of 4-5 independent enzyme purifications.

Figure S5. **Biofilm synthesis in *M. acetivorans***

Cells were cultured in acetate with different Cd^2+^ concentrations and the biofilm synthesis was determined as indicated in the methods section. Data are the mean ± SD of 5-7 independent experiments*.* ^a^ P<0.001 *vs.* control, ^b^ P<0.025 *vs.* control cells.

Figure S6. **Sequence alignments of PPK and PPX of *M. acetivorans***

PPK alignment gene ID 1471973 of *M. acetivorans* with other protein sequences of different microorganisms **(A)**: Ecoli*= Escherichia coli*; Cpse= *Corynebacterium pseudodiphtheriticum*; Mprof= *Methanolobus profundi*; Asp= *Acidithiobacillus sp.* CJ-2; Aferroox= *Acidithiobacillus ferrooxidans*. **(B)** PPX alignment gene ID 1471975 of *M. acetivorans* with protein sequences of different microorganisms: Msic= *Methanosarcina siciliae*; Ecoli= *Escherichia coli*; Csp= *Corynebacterium sp*.; Mprof= *Methanolobus profundi*; Ctep= *Chlorobaculum tepidum.* Alignments were performed using Clustal W2 and Clustal O (1.2.4) for PPK and PPX, respectively. Asterisks (*) indicate homologous amino acids; colons (:) indicate conserved substitutions; periods (.) indicate semi-conserved substitutions.
